# Clinical Outcomes Associated With His-Purkinje System Pacing vs. Biventricular Pacing, in Cardiac Resynchronization Therapy: A Meta-Analysis

**DOI:** 10.3389/fcvm.2022.707148

**Published:** 2022-02-11

**Authors:** Yang Gui, Lifang Ye, Liuyang Wu, Haohui Mai, Qiqi Yan, Lihong Wang

**Affiliations:** ^1^BengBu Medical College, Bengbu, China; ^2^Department of Cardiovascular Medicine, Zhejiang Provincial People's Hospital, People's Hospital of Hangzhou Medical College, Hangzhou, China

**Keywords:** cardiac resynchronization therapy, His-Purkinje system pacing, biventricular pacing, meta-analysis, biventricular pacing, meta-analysis (as topic)

## Abstract

**Aims:**

His-Purkinje system pacing has recently emerged as an alternative to biventricular pacing (BIVP) in cardiac resynchronization therapy (CRT). The aim of this study was to conduct a meta-analysis comparing the clinical outcomes associated with His-Purkinje system pacing (HPSP) vs. BIVP in patients with heart failure. There is also a comparison of clinical outcomes of His-bundle pacing (HBP) and left bundle branch pacing (LBBP) in the His-Purkinje system.

**Methods:**

We searched the Cochrane Library, Embase, and PubMed, for studies published between January 2010 and October 2021 that compared the clinical outcomes associated with HPSP vs. BIVP and HBP vs. LBBP in HPSP in patients who underwent CRT. The pacing threshold, R-wave amplitudes, QRS duration, New York Heart Association functional (NYHA), left ventricular ejection fraction (LVEF), and LV end-diastolic diameter (LVEDD) of heart failure, at follow-up, were extracted and summarized for meta-analysis.

**Results:**

A total of 18 studies and 1517 patients were included in our analysis. After a follow-up period of 9.3 ± 5.4 months, the HPSP was found to be associated with shorter QRS duration in the CRT population compared to that in the BIVP (SMD, −1.17; 95% CI, −1.56 to −0.78; *P* < 0.00001; I^2^ = 74%). No statistical difference was verified between HBP and LBBP on QRS duration (SMD, 0.04; 95% CI, −0.32 to 0.40; *P* = 0.82; I^2^ = 84%). In the comparison of HPSP and BIVP, the LBBP subgroup showed improved LVEF (SMD, 0.67; 95% CI, 0.42–0.91; *P* < 0.00001; I^2^ = 0%), shorter LVEDD (SMD, 0.59; 95% CI, 0.93–0.26; *P* = 0.0005; I^2^ = 0%), and higher New York Heart Association functional class (SMD, −0.65; 95% CI, −0.86 to −0.43; *P* < 0.00001; I^2^ = 45%). In terms of pacing threshold and R-wave amplitude clinical outcomes, LBBP has a lower pacing threshold (SMD, 1.25; 95% CI, 1.12–1.39; *P* < 0.00001; I^2^ = 47%) and higher R-wave amplitude (MD, −7.88; 95% CI, −8.46 to −7.31; *P* < 0.00001; I^2^ = 8%) performance compared to HBP.

**Conclusion:**

Our meta-analysis showed that the HPSP produced higher LVEF, shorter QRS duration, and higher NYHA functional class in the CRT population than the BIVP as observed on follow-up. LBBP has a lower pacing threshold and higher R-wave amplitude. HPSP may be a new and promising alternative to BIVP in the future.

## Highlights

- QRS duration was shorter in His-Purkinje system pacing than in biventricular pacing.
- The left bundle branch pacing group in His-Purkinje system pacing is associated with improved LVEF, increased LVEDD, and higher NYHA functional class.
- In patients with heart failure who underwent cardiac resynchronization therapy, the His-Purkinje system pacing showed better results than biventricular pacing.
- LBBP has a lower pacing threshold and higher R-wave amplitude.

## Introduction

Cardiac resynchronization therapy (CRT) is used to treat patients with heart failure (HF), and ventricular systolic dyssynchrony. By electrically activating the heart in a coordinated manner, CRT can successfully restore mechanical synchrony. Traditionally, this therapy has been implemented using biventricular pacing. Studies have shown that biventricular pacing (BIVP) can improve symptoms, reduce hospitalization times, and prolong the survival of patients (1–4). However, multiple clinical trials have demonstrated that 30-40% of patients showed no changes after BIVP-based CRT (5–10).

In 2015, a crossover study by Lustgarten et al. showed that His-bundle pacing (HBP) can achieve clinical outcomes comparable to BIVP (11). Similarly, several other studies have suggested that HBP may be a suitable alternative for CRT non-responders and patients with failed left ventricle (LV) lead placement (12–14); some of these studies have even recommended HBP as frontline therapy for heart failure and left ventricle dyssynchrony (12–14). In addition, recent guidelines by the American College of Cardiology/American Heart Association have assigned HBP a grade II in terms of recommendation for replacing right ventricular pacing in patients who need chronic ventricular pacing with reduced LV ejection fraction (LVEF; 36–50%) (11, 15). More recently, however, studies compared HPSP with BIVP pacing and evaluated the potential advantages in CRT. The HPSP is characterized by a generation of strategies that can mimic pacing or fully restore normal atrioventricular (AV) activation, ensuring optimal clinical outcomes; it involves left bundle branch pacing (LBBP) and HBP. LBBP can correct left bundle branch blocks (LBBB) and, thus, lead to improvement of cardiac electrical dyssynchrony compared with conventional right ventricular apical pacing (16). LBBP produces a lower pacing capture threshold and higher R-wave amplitude than HBP and stimulates the conduction system of the heart as well as the deep septal myocardium (17, 18). The role of His-Purkinje conduction system is usually to produce true cardiac resynchronization. In contrast, some studies have concluded that ventricular mechanical synchronization parameters are significantly better in patients with HBP than in patients with right ventricular septal pacing (RVSP) (19, 20).

HBP is the most physiological pacing strategy for restoring normal ventricular excitation patterns (21). In the case of His bundle pacing (HBP), HBP corrects complete left bundle branch block (CLBBB) by activating the heart's intrinsic conduction system and thus providing natural ventricular excitation propagation (22, 23). There are currently no publications that comprehensively analyze and summarize the data generated from clinical trials that have evaluated the influence of HPSP therapy. Currently for the His-Purkinje conduction system, both the comparison with conventional BIVP pacing and the advantages and disadvantages of HBP vs. LBBP pacing in the His-Purkinje conduction system have a great role for CRT. Therefore, this study aimed to compare HPSP and BIVP in clinical outcomes in patients with HF and to conduct a meta-analysis.

## Methods

This study protocol has been published previously in PROSPERO (CRD42021235736).

### Search Strategy

The meta-analysis was conducted according to the meta-analysis statement and the preferred reporting items for systematic reviews (24). We selected relevant studies published between January 2010 and October 2021 by searching PubMed, EMBASE, and Cochrane Library. Our search did not have any language restrictions. The search terms were “His bundle pacing” OR “Left branch bundle pacing” OR “biventricular pacing” AND “Cardiac Resynchronization Therapy.” In addition, we also searched the list of references in the studies retrieved by our search criteria.

### Study Eligibility Criteria

We included randomized clinical trials (RCTs) and observational studies which examined patients with HF requiring CRT. Specifically, studies were included if they (i) were RCTs, (ii) were observational studies, or (iii) reported empirical data regarding clinical outcomes, including Pacing threshold, R-wave amplitudes, QRS duration, LVEF, LV end-diastolic diameter (LVEDD), and New York Heart Association (NYHA) class of HF. Studies were excluded if they (i) were missing text, (ii) reported results from a previously included study, (iii) did not include or directly study CRT, or (iv) had missing data or insufficient original data.

### Data Extraction

Two reviewers independently extracted data from the included RCTs and observational studies; disagreements were resolved by consensus through discussion. We recorded the following information from the included RCTs and observational studies: duration of follow-up, number of participants, and year of publication, and study design. We also extracted information on pacing threshold, R-wave amplitudes QRS duration, LVEF, LVEDD, and NYHA HF class.

### Quality Assessment

Two reviewers independently assessed the RCTs included in this study using the Jadad scoring system (25), which assesses the methodological quality of RCTs. Investigations that received Jadad scores below 4 (out of a possible 5) were classified as low-quality, while those that scored ≥4 were deemed high-quality. Among the included observational studies, for the retrospective studies and cohort studies, assessment of using the Newcastle Ottawa scale (NOS) (26) to performed the quality of nonrandomized studies. Investigations that received NOS scores below 6 (out of a possible 9) were classified as low-quality, while those that scored ≥6 were deemed high-quality. When the format of the required data for inclusion was not suitable for the meta-analysis, the primary authors and publishing journals were contacted by email to access unpublished data.

### Statistical Analyses

For all statistical analyses, RevMan 5.3 software (27) was used. A comprehensive analysis of individual studies was done to compare the different effects of His-Purkinje system pacing and BIVP in patients with HF. We assessed statistical heterogeneity with the Q statistic from the chi-square test and *P* < 0.05 represented a significant result. We dequantified the proportion of variation using the I^2^ statistics between studies due to heterogeneity. It was considered that there was little heterogeneity between studies if *P* ≥ 0.1, or I^2^ ≤ 50%; *P* < 0.1, or I^2^ > 50% indicated moderate heterogeneity, and I^2^ > 75% indicated considerable heterogeneity, I^2^ ≤ 50% used fixed-effects model and I^2^ > 50% used-random effects model. A subgroup analysis was attempted to find the source of heterogeneity. To analyze the literature for the presence or absence of publication bias, we used funnel plots. The mean and standard deviation were reported for continuous variables. Review Manager V5.3 (27) was used for all data processing analyses.

## Results

### Study and Patient Characteristics

**Figure 1:**
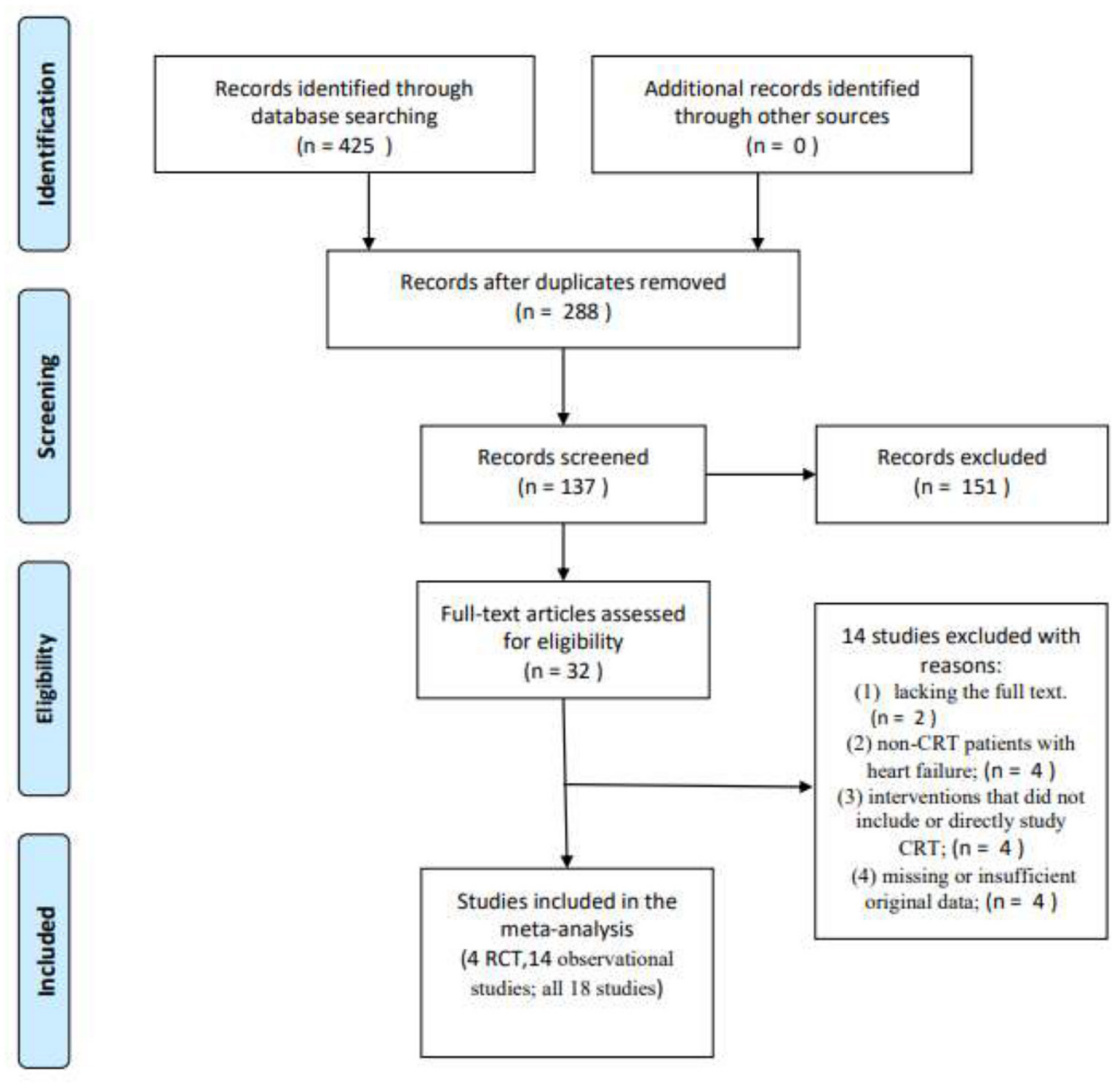
A flow diagram showing how articles were selected for analysis, Moher et al. (28).

**Figure 2:**
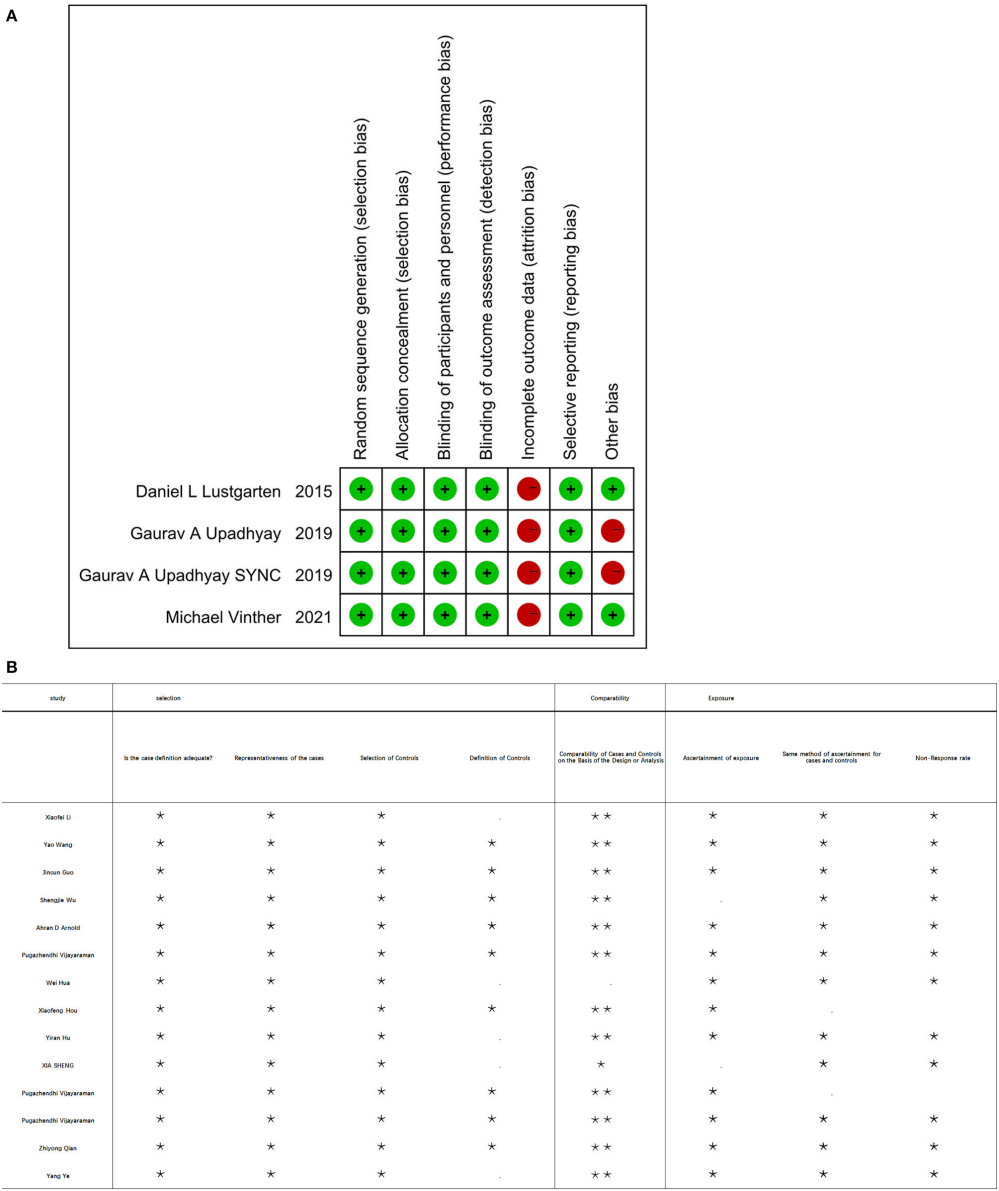
**(A)** Four of the included RCT studies were using scoring system at risk of bias. **(B)** Fourteen of the included studies using the Newcastle Ottawa scale (NOS).

Initially, a total of 425 articles were retrieved. Out of which, 32 articles were retained for full article evaluation by reviewing the study titles with the abstracts. Duplicate reviews and duplicate case reports with non-relevant studies were excluded. These 32 studies underwent a thorough screening process as shown in Figure 1. Following the screening, 18 studies were included in our analysis; four of these were RCT studies, while 14 were observational studies. Ten of them are the comparison of HPSP with BIVP and eight are the comparison of HBP with LBBP in HPSP. Further details regarding the studies analyzed are shown in Table 1. The 18 included studies (11, 29–45), which were RCTs and observational studies, were scored using the Jadad scoring system and the NOS quality assessment system, as shown in Figures 2A,B.

**Table 1:** Basic characteristics of included studies analyzed during this study.

**References**	**Type of study**	**Age (year)**	**QRSd**	**LVEF**	**Male (%)**	**Region**	**Period**	**Number of** **patients** **(physiologic/** **BiVP)**	**Indication of pacing**	**Pacing** **sites**	**Follow-up months**	**Evaluated parameters**
Li et al. (29)	Observational	56.8 ± 10.1	177.9 ± 18.8	29.3 ± 5.9	59.5	China	2020	27/54	LBBB (LVEF) ≤ 35%	LBBP BiVP	6 month	QRSd LVEF NYHA LVEDD
Wang et al. (30)	Observational case-control	63.4 ± 9.6	176.9 ± 19.6	26.5 ± 4.9	0.8	China	2020	10/30	HF LVEF ≤ 35% NYHA2-4	LBBP BiVP	6 month	QRSd LVEF NYHA LVEDD LVESV LVESD
Guo et al. (31)	Prospective observational	65.6 ± 8.6	165.7 ± 14.3	29.9 ± 4.5	0.428	China	2020	21/21	HF LBBB	LBBP BiVP	14.3 ± 7.2 month	QRSd LVEF NYHA LVEDD
Wu et al. (32)	Non-randomized observational	67.9 ± 11.1	163 ± 11.5	30.7 ± 6.6	0.5	China	2020	32/54	LVEF ≤ 40% LBBB	LBBP BiVP	12 month	QRSd LVEF NYHA LVESV LVESD
Lustgarten et al. (11)	Randomized controlled trial	71.33	169 ± 16	26 ± 55.6	0.66	Burlington	2015	29 (12/12)	QRSd > 130 ms	HBP BiVP	6 month	QRSd LVEF NYHA LVESV LVESD 6-min walk
Upadhyay et al. (33)	Randomized controlled trial	64 6 13	168.6 ± 18	28	0.62	Chicago	2019	21/20	HF	HBP BiVP	12 month	QRSd LVEF
Arnold et al. (34)	Observational	67 ± 10	158 ± 21	26 ± 7	0.53	British	2018	23/23	QRSd > 130 ms LVEF ≤ 35% NYHA2-4	HBP BiVP	12 month	QRSd
Vijayaraman et al. (35)	Observational	72 ± 15	183 ± 27	24 ± 7	0.85	Florida	2019	10/16	LVEF ≤ 40% LBBB	HBP BiVP	14 ± 10 month	QRSd LVEF NYHA LVEDD
Upadhyay et al. (36)	Randomized controlled trial	64 ± 13	168 ± 18	28	0.62	Chicago	2019	21/20	HF	HBP BiVP	12 month	QRSd LVEF
Vinther et al. (37)	Randomized controlled trial	65.8 ± 9.3	166 ± 15	30 ± 7	0.64	Denmark	2021	25/25	LVEF <35, HF, LBBB	HBP BiVP	6 month	LVEF PT LVESV NYHA
Hua et al. (38)	Observational study	63.8 ± 13.4	108.6 ± 23.8	58 ± 7.7	0.51	China	2020	109/115	Symptomatic bradycardia	HBP LBBP	3 month	QRSd PT R-wave
Hou et al. (39)	Single-centre prospective	68.6 ± 11.3	105.8 ± 26.4	63.6 ± 4.2	0.647	China	2019	29/56	SND AVB (atrioventricular block)	HBP LBBP	4.5 ± 2.4 month	QRSd LVEF R-wave PT
Hu et al. (40)	Prospective, observational, nonrandomized	61.4 ± 18.1	119 ± 16.2	57.5 ± 9.5	0.64	China	2020	25/25	AVB	HBP LBBP	3 month	QRSd LVEF LVEDD R-wave PT
Sheng et al. (41)	Single-center prospective patient control	72.9 ± 9.0	96.5 ± 16.2	62 ± 12	0.654	China	2021	10/10	AF with slow ventricular rate	HBP LBBP	3 month	QRSd PT R-wave
Vijayaraman et al. (42)	Prospective, single-center observational study	75.7 ± 22	121 ± 30	53.5 ± 22.7	0.63	Florida	2021	143/182	AVB	HBP LBBP	24 month	QRSd PT R-wave
Vijayaraman et al. (43)	Observational retrospective	79 ± 8	138.7 ± 28.8	58 ± 12	0.57	Florida	2020	29/26	AVCD after TAVR	HBP LBBP	12 ± 13.7	QRSd PT R-wave LVEF
Qian et al. (44)	Single-centre observational	68.3 ± 12.1	142.3 ± 30.7	63 ± 53.8	0.562	China	2020	64/185	HF	HBP LBBP	12 month	QRSd PT R-wave LVEF
Ye et al. (45)	Non-controlled non-randomized prospective	78 ± 5	91 ± 10	35.1 ± 11.7	0.75	China	2020	14/13	AF	HBP LBBP	6 month	QRSd PT R-wave LVEF

*AF, atrial fibrillation; AVB, atrioventricular block; AVCD, AV conduction disease; HF, heart failure; QRSd, QRS duration;#LVEF, left ventricular ejection fraction; LVEDD, left ventricular end-diastolic dimension; PT, pacing thresholds; R-wave, R-wave amplitudes; NYHA, New York Heart Association; HBP, His-bundle pacing; LBBP, left bundle branch pacing, BIVP, biventricular pacing*.

### QRS Duration

**Figure 3:**
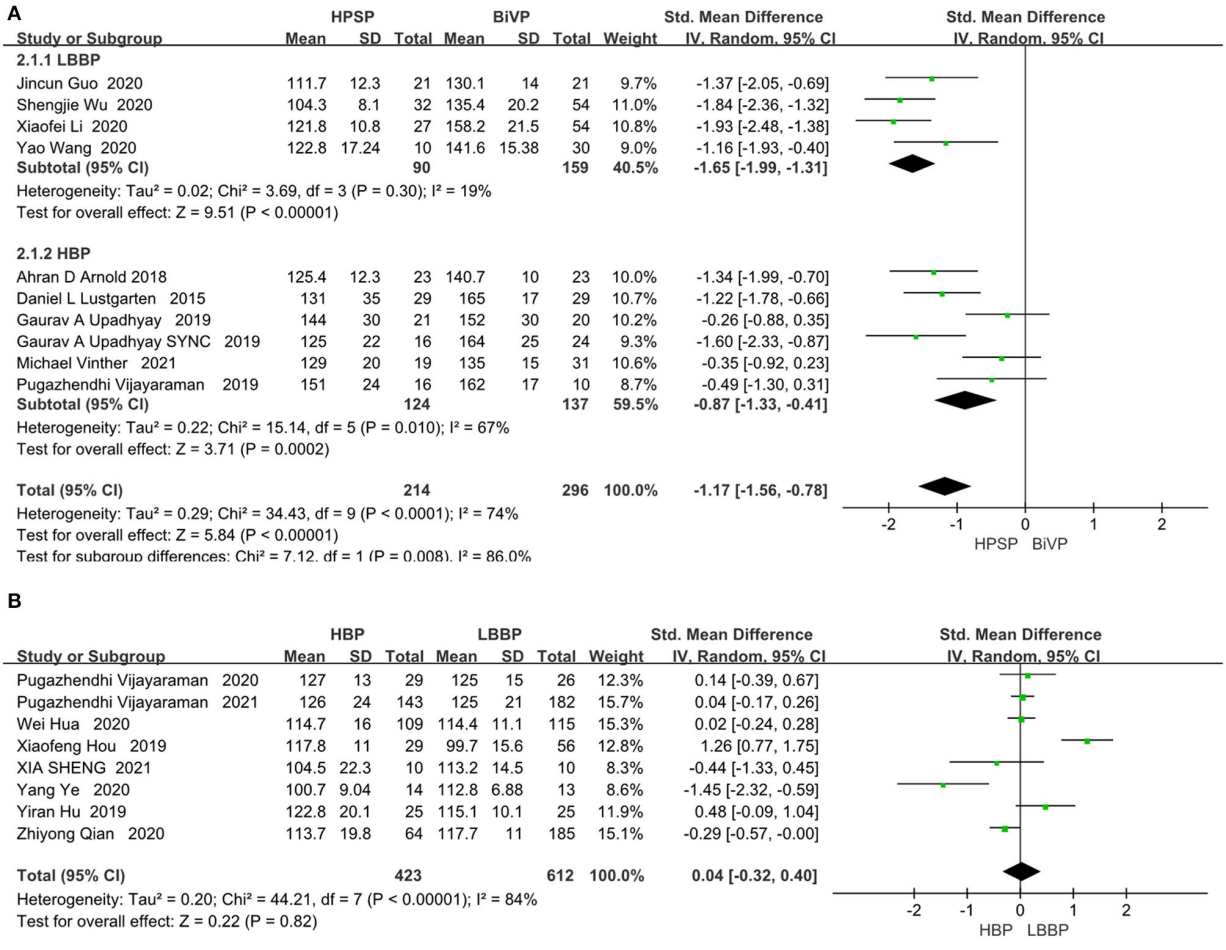
**(A)** QRS duration in patients receiving HPSP therapy vs. BIVP therapy. **(B)** QRS duration in patients receiving LBBP vs. HBP [**(A)** top table; **(B)** bottom table].

The heterogeneity between individual studies was tested by analyzing differences in the QRS duration in 482 patients from 10 studies (I^2^ = 74%). The random-effect model was used. As shown in Figure 3A, patients treated with the His-Purkinje system pacing had shorter QRS duration than those treated with BIVP (SMD, −1.17; 95% CI, −1.56 to −0.78; *P* < 0.00001; I^2^ = 74%; Figure 3A). Although the heterogeneity test between the 10 studies indicated that there was moderate heterogeneity, sensitivity analysis showed that the results did not change significantly among all the studies included.

The eight included papers on HBP and LBBP directly compared clinical outcomes. There was no significant difference between LBP and LBBP in the QRS duration index (SMD, 0.04; 95% CI, −0.32 to 0.40; *P* = 0.82; I^2^ = 84%; Figure 3B). HPSP produced a reduction in QRS duration compared to the BIVP group, but no differences were found when comparing within groups.

### LV Function Assessment

**Figure 4:**
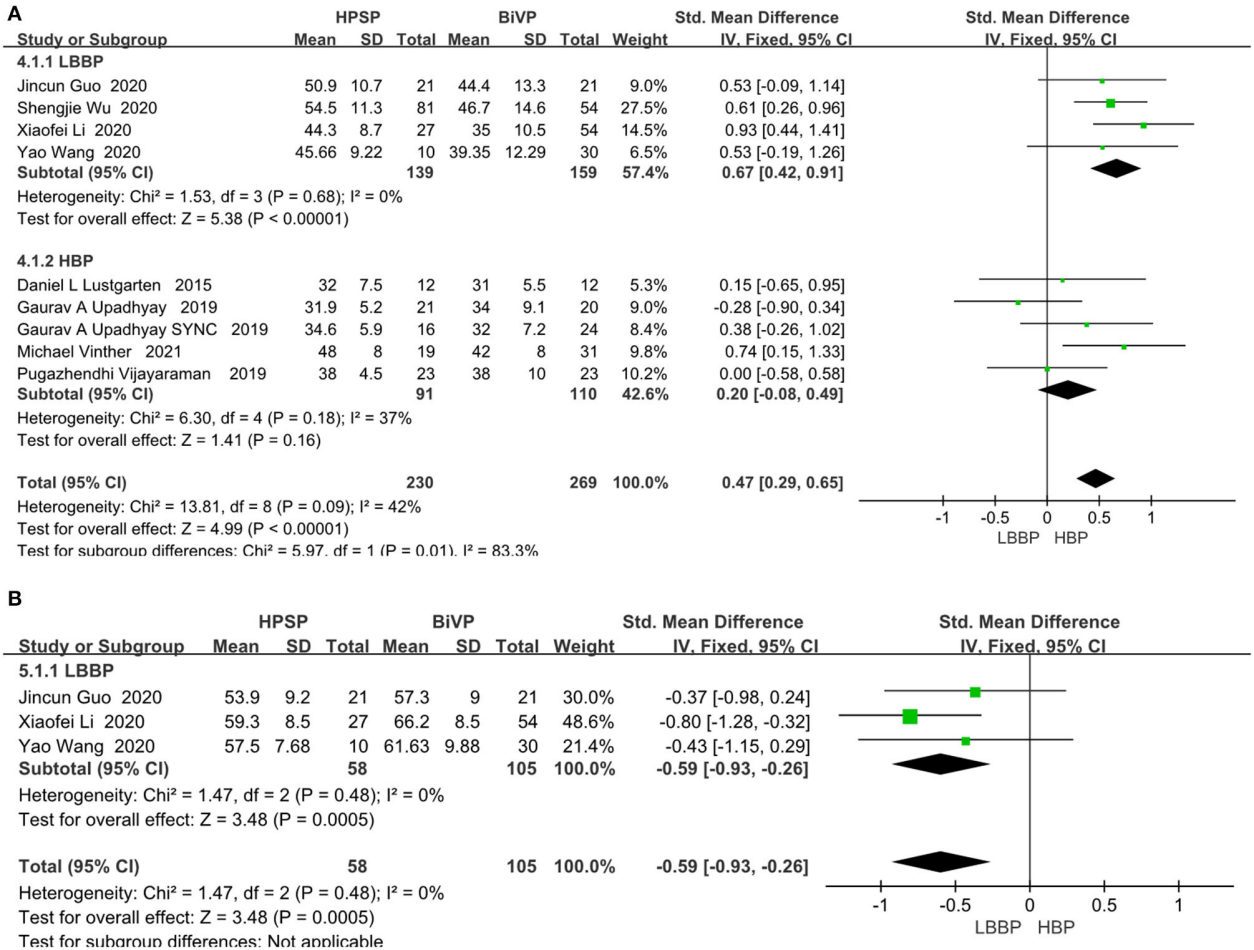
**(A)** LVEF. **(B)** LVEDD. Left ventricular function pacing of the His-Purkinje system is required in patients with HF therapy vs. biventricular pacing therapy. Both LVEF and LVEDD were measured by echocardiography.

LVEF was analyzed by fixed models in 436 patients from nine studies. The LVEF fraction was higher in the HPSP group, compared with that in the BIVP group (SMD, 0.47; 95% CI, 0.29–0.65; *P* < 0.00001; I^2^ = 42%; Figure 4A).There was little heterogeneity among the study results (*P* < 0.00001; I^2^ = 42%). Three studies were included in the evaluation of LVEDD differences. We used the fixed-effects model because of the heterogeneity between the studies (I^2^ = 0%). When compared with BIVP, the His-Purkinje system pacing indicated better performance (SMD, 0.59; 95% CI, 0.93–0.26; *P* = 0.0005; I^2^ = 0%; Figure 4B).

### NYHA Functional Class

**Figure 5:**
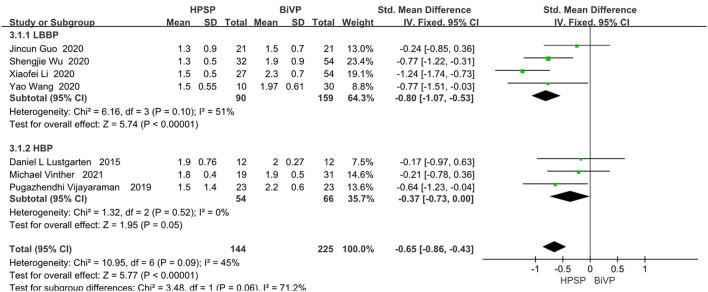
New York Heart Association functional class in patients receiving His-Purkinje system pacing therapy vs. biventricular pacing therapy.

Of the eight included studies, seven of them reported a functionally relevant improvement analysis. We used the random-effect model because of the heterogeneity between the studies (I^2^ = 45%). Compared with BIVP, His-Purkinje system pacing indicated better performance (SMD, −0.65; 95% CI, −0.86 to −0.43; *P* < 0.00001; I^2^ = 45%, Figure 5). No evidence of publication bias was found, after passing the inspection of the corresponding funnel plots.

#### Pacing Threshold

**Figure 6:**
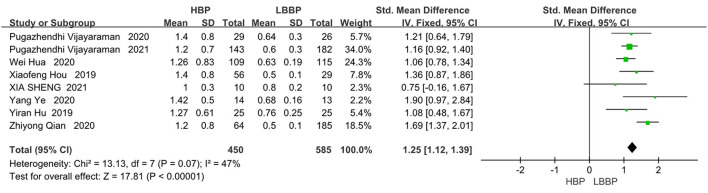
Pacing thresholds in patients receiving comparison between HBP and LBBP in His-Purkinje system.

In the eight papers we adopted on the direct comparison between LBBP and HBP, the pacing threshold indexes all showed a great advantage of LBBP (SMD, 1.25; 95% CI, 1.12–1.39; *P* < 0.00001; I^2^ = 47%, Figure 6).

#### R-wave Amplitudes

**Figure 7:**
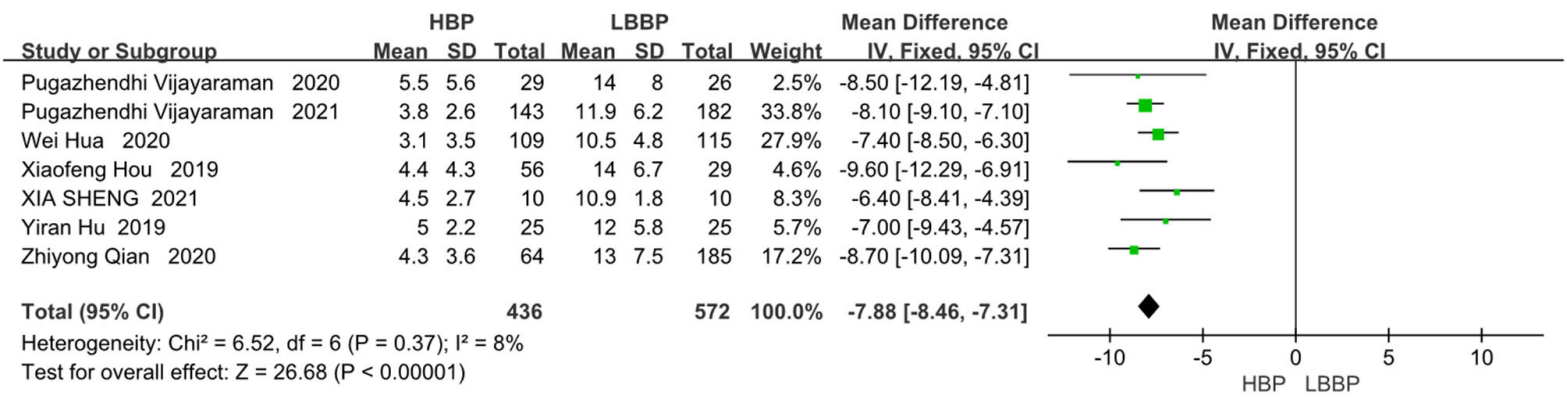
R-wave amplitudes in patients receiving comparison between HBP and LBBP in His-Purkinje system.

Seven of the eight included papers reported R-wave amplitudes, with LBBP reflecting considerable R-wave amplitudes compared to HBP (MD, −7.88; 95% CI, −8.46 to −7.31; *P* < 0.00001; I^2^ = 8%, Figure 7).

## Discussion

This systematic review and meta-analysis identified 18 trials with a total of 1,517 participants and compared cardiac electrophysiology and cardiac function in HPSP and BIVP and in HBP and LBBP. Ultimately, we concluded that HPSP resulted in a favorable improvement in QRS duration in patients with HF, while LBBP improved LV function and improved NYHA functional class in CRT candidates. When HBP and LBBP were directly compared in terms of the His-Purkinje system, LBBP demonstrated a lower pacing threshold and higher R-wave amplitude than HBP.

Several randomized controlled trials and observational studies have shown that long-term differences in LVEF have the potential to lead to interventricular dyssynchrony. One of the parameters of interventricular dyssynchrony is QRS duration (29–33, 35, 46). In the present study, the HPSP group performed better than the BIVP group in terms of QRS duration. It can also be argued that LBBP or HBP may produce better electromechanical synchronization and thus induce more synchronized LV contractions. In our study, HPSP improved the QRS duration by 22.23 ms relative to BIVP. Moreover, no difference in QRSd was found between LBBP and HBP (*P* = 0.82).

Sheng et al. (41) also confirmed that HBP and LBBP produce similar QRSd. During atrial fibrillation, LBBP is equally as viable as HBP. A unique finding of Sheng's (41) study was the difference in interventricular synchrony between HBP and LBBP. In contrast, the unipolar configuration of LBBP produced a slightly later contraction of the right ventricular myocardium compared to that produced by HBP. In bradycardic patients requiring CRT, HBP and LBBP led to similar QRSd and implantation success rates and shorter procedure and fluoroscopy times. However, the study (41) also noted a significantly lower pacing threshold for LBBP and a higher R-wave amplitude at implantation and at the 3-month follow-up. Moreover, LBBP has better clinical feasibility compared to the HBP. This is consistent with our findings comparing HBP with LBBP, in which LBBP improved pacing thresholds by an average of 0.62 ms over HBP and by 7.88 mv in R-wave amplitude. Chen et al. (47) demonstrated the clinical feasibility of LBBP by using a transventricular septal approach. Massing et al. (48) suggested that LBBP could directly branch out from the branch point of the His bundle in the cardiac structure under the endocardium on the left side of the septum, thus forming a reticular structure, so that the left bundle branch can be paced faster than by HBP through the septal approach. This may explain the better pacing threshold and R-wave amplitude of LBBP compared with HBP. Zhang et al. (49) attributed the narrow QRS pattern during LBBP to the activation of the right bundle branch of the ventricle by electrophysiological retrograde conduction, which forms a connection with intrinsic conduction fusion. Huang et al. (50) had a higher success rate and a stable lower pacing threshold with LBBP than HBP and a better perception of ventricular excitation (R-wave amplitude).

LBBP is now the preferred conduction system pacing modality for patients with pacing indications (20, 21). Li et al. (21) reported on LBBP in 33 patients with AVB and found that it has a success rate of more than 90%, produces low and stable thresholds, maintains LV synchronization, and has few complications. The current potential hypothesis is that LBBP further enriches physiological pacing and may even be more applicable to patients with AVB. Furthermore, Vinther et al. (37) found that His bundle improved ventricular function and quality of life, but this was at the cost of a higher pacing threshold. Hou et al. (39) found that left bundle branch pacing produced higher R-wave amplitude than HBP and lower capture threshold stability parameters than HBP. Qian et al. (44) concluded that His-Purkinje system pacing produces good electrical synchronization and narrow QRS time frames and that it has beneficial effects in maintaining cardiac function. In contrast, left bundle branch pacing showed superior lead stability in terms of pacing parameters. Ye et al. (45) found that both HBP and LBBP can be successfully implemented in the same patient with atrial fibrillation and that LBBP produces better and more stable parameters compared to HBP. Patients with AF with HF and arrhythmias benefit more from HPSP in terms of physical performance and echocardiographic parameters.

Overall, we concluded that HPSP produced better electromechanical synchronization than BIVP; further, when comparing HPSP within groups, LBBP had higher success rates, lower pacing thresholds, and higher R-wave amplitudes compared to HBP.

HPSP, a physiological pacing modality that directly stimulates the conduction system of the heart and maintains synchronization of ventricular electrical activation has produced better results compared to BIVP in clinical practice (41, 45). Lustgarten et al. (11) summarized the clinical outcome data from a 2015 study of 12 patients with a mean baseline LVEF of 26%; at the 6-month follow-up, HBP was shown to improve by 32% and BIVP by 31% (*P* = 0.043 and *P* = 0.02, respectively); the baseline NYHA grades for HBP and BIVP improved from 2.9 to 1.9 (*P* < 0.01 and *P* < 0.01, respectively). The multicenter 2019 RCT His-SYNC study by Upadhyay et al. (33) included 41 patients from 7 centers who met the criteria indications for CRT; 20 and 21 of these patients were randomized to the BIVP CRT and His CRT groups, respectively. Patients in both groups showed a significant improvement in LVEF after 6.2 months of follow-up, when compared with the baseline values. The median LVEF increased from 28.0 to 34.6% (*P* < 0.001) in patients treated with HBP CRT, whereas it increased from 27.7 to 32.0% (*P* < 0.001) in those treated with BIVP CRT. To determine the difference in LV function by pacing modality, we also compared LVEF, LVEDD, and NYHA. In our meta-analysis, LVEF was significantly improved in both groups compared with the baseline values at the 6-month follow-up. HPSP showed a 3.91% improvement in LVEF, a 5.36 mm reduction in LVEDD, and a 0.44 grade reduction in NYHA compared with BIVP. Clinical outcomes were similar for BIVP and HBP. In patients with HF, cardiac resynchronization can be achieved by pacing the His-Purkinje system to correct LBBB. Theoretically, HPSP may be more physiologically consistent than BIVP because the latter still relies on stimuli that do not propagate through the normal conduction system but through the myocardium. The relatively small number of 18 studies analyzed may have influenced the results. Larger RCTs are needed to validate the relationship between His-Purkinje system pacing and BIVP.

In summary, we conclude that the His-Purkinje system produces higher LVEF, shorter QRS duration, and higher NYHA functional class in the CRT group compared to BIVP in pacing therapy overall. When comparing HPSP systems within groups, LBBP had a higher success rate, a lower pacing threshold, and higher R-wave amplitude compared to HBP. HPSP may be a new and promising alternative to BIVP in the future.

### Study Limitations

This meta-analysis has several limitations. First, is a bias due to the small number of included relevant RCTs and the fact that most studies (29–32, 34, 35, 38–45) were *post-hoc* analyses. This bias may have influenced the conclusions of the present study. Second, the length of follow-up in the included literature takes longer to justify the results. Third, this study did not include data on mortality or cardiovascular hospitalization. Fourth, the complications after different pacing procedures are not discussed.

## Conclusion

In conclusion, the HPSP can produce shorter QRS duration, higher LVEF, and higher NYHA functional class in the CRT population compared with BIVP as observed by follow-up. HPSP may be a new and promising alternative to BIVP in the future. LBBP has a lower pacing threshold and higher R-wave amplitude. Considering the clinical significance of pacing therapies, RCTs are required to further evaluate the efficacy of HPSP compared with BIVP in achieving CRT.

## Data Availability Statement

The original contributions presented in the study are included in the article/supplementary material, further inquiries can be directed to the corresponding author.

## Author Contributions

All authors listed have made a substantial, direct, and intellectual contribution to the work and approved it for publication.

## Funding

This study was supported by the National Natural Science Foundation of China under Grant No. 81670447; the National Natural Science Foundation of Zhejiang Province under Grant No. LY15H020006; Zhejiang Province Key Subject of Medicine (Neurological Rehabilitation) and the Traditional Chinese Medicine Program of Zhejiang Provincial under Grant No. 2017ZZ001; the Zhejiang Provincial Health Commission Project under Grant No. 2017KY559; the National Natural Science Foundation of Zhejiang Province under Grant No. LY19H070003. LW is sponsored by Zhejiang Provincial Program for the Cultivation of High-Level Innovative Health Talents.

## Conflict of Interest

The authors declare that the research was conducted in the absence of any commercial or financial relationships that could be construed as a potential conflict ofinterest.

## Publisher's Note

All claims expressed in this article are solely those of the authors and do not necessarily represent those of their affiliated organizations, or those of the publisher, the editors and the reviewers. Any product that may be evaluated in this article, or claim that may be made by its manufacturer, is not guaranteed or endorsed by the publisher.
